# The Transition From Ambrisentan to Macitentan in Patients With Pulmonary Arterial Hypertension: A Real-word Prospective Study

**DOI:** 10.3389/fphar.2021.811700

**Published:** 2022-01-12

**Authors:** Yusi Chen, Jun Luo, Jingyuan Chen, Eugene Kotlyar, Zilu Li, Wenjie Chen, Jiang Li

**Affiliations:** ^1^ Department of Cardiovascular Medicine, The Second Xiangya Hospital of Central South University, Changsha, China; ^2^ St. Vincent’s Hospital, Sydney, NSW, Australia

**Keywords:** pulmonary arterial hypertension, macitentan, ambrisentan, endothelin receptor antagonist, real-word study

## Abstract

**Background:** In a long-term event-driven trial, macitentan has demonstrated beneficial time to clinical worsening in patients with pulmonary arterial hypertension (PAH) and reduced PAH-related hospitalization rates compared with placebo. Macitentan is the most recently approved endothelin receptor antagonist (ERA) and is the first ERA that has shown efficacy for morbidity and mortality in PAH patients; therefore, patients and physicians may consider converting treatment from ambrisentan to macitentan. Our study evaluated the safety, efficacy, and quality of life in PAH patients transitioning from ambrisentan to macitentan.

**Methods:** This was a real-world, prospective study with a 12-month follow-up. PAH patients who had received stable doses of ambrisentan for over 3 months, were within the World Health Organization Functional Class II/III, and 6-min walk distance ≥ of 250 m were enrolled. The study included a screening period, followed by a transition phase, after which patients entered the long-term follow-up. Clinical data and treatment satisfaction outcomes were collected to assess and monitor the safety and efficacy of the transition. The trial was registered at the Chinese Clinical Trial Registry (www.chictr.org.cn; No. ChiCTR2000034898).

**Results:** One hundred and fifty-seven enrolled PAH patients completed the transition. All criteria for continuous treatment transition were met by 145 patients (92.4%). Results showed improvements in exercise capacity, cardiac function, and hemodynamics compared with baseline. During the process, 4 patients discontinued macitentan due to adverse events. There was no statistical difference in the overall incidence of adverse events before and after the transition.

**Conclusion:** Transition to macitentan from ambrisentan was successful and well-tolerated by PAH patients, and was associated with greater efficacy and satisfaction.

## Introduction

Pulmonary arterial hypertension (PAH) is a progressive pulmonary vascular disease associated with high mortality, characterized by pulmonary vascular remodeling and increased pulmonary vascular resistance (PVR) (Boucherat et al., 2017). Without timely treatment, PAH eventually leads to right ventricular failure and even death. Over the past several decades, advances in targeted therapies have significantly improved the prognosis of PAH patients. Medications targeting the endothelin pathway play an important role in the treatment of PAH. Endothelin 1 (ET-1), a potent vasoconstrictor that modulates pulmonary vascular remodeling, is overexpressed within the remodeled pulmonary arteries and results in vascular narrowing (Benza et al., 2015a; Dai et al., 2019). ET-1 binds to two receptors, endothelin type A (ET-A) and endothelin type B (ET-B) to mediate its effects. Endothelin receptor antagonists (ERA) that inhibit either ET-A or ET-A and ET-B receptors are used as monotherapy or as a component of combination regimens to treat PAH.

At present, approved ERA medications for PAH include bosentan, ambrisentan, and macitentan, all of which are available in China. Bosentan was the first available ERA; however, it has been shown to increase the incidence of dose-dependent increases in liver aminotransferase concentrations (Markova et al., 2013). More recently, two other ERAs, macitentan and ambrisentan, which have rare hepatotoxicity, have been approved for the treatment of PAH.

Ambrisentan, the only available selective ERA, is developed theoretically to inhibit the vasoconstrictor function of ET-A while not damaging the vasodilatory and clearance effects of ET-B (Hitzerd et al., 2020). The ARIES-1 (Ambrisentan in Pulmonary Arterial Hypertension, Randomized, Double-Blind, Placebo-Controlled, Multicenter, Efficacy Study) and ARIES-2 trials had shown that ambrisentan alleviates exercise intolerance and improves World Health Organization Functional Class (WHO FC) and hemodynamics in PAH patients (Galiè et al., 2008) after 12 weeks of therapy. Furthermore, the AMBITION trial demonstrated that an upfront combination of ambrisentan with tadalafil, a phosphodiesterase type 5 inhibitor (PDE-5i), was associated with a 63% reduction in risk of PAH-related hospitalization compared with monotherapy (Vachiéry et al., 2019) and delayed clinical deterioration in PAH when used initially (Hoeper et al., 2016). However, no large-scale studies to date have demonstrated that ambrisentan monotherapy results in improved long-term morbidity and mortality in PAH patients (Oudiz et al., 2009; Hoeper et al., 2016).

Macitentan is the most recently developed ERA to be approved for management of PAH, and has favorable pharmacokinetics, without necessitating monthly monitoring hepatic risk. In the early stages of research and development, macitentan exhibited better tissue penetration and receptor affinity than did bosentan and ambrisentan. In an event-driven 100 week-long trial, compared with placebo, macitentan demonstrated a significant reduction in clinical worsening in PAH, first PAH-related events, or all-cause mortality in patients with WHO FC II and III, and significantly reduced PAH-related hospitalization rates (Pulido et al., 2013). Macitentan is the first ERA that has been approved to reduce morbidity and mortality in patients with PAH. SERAPHIN found that among the 64% of PAH patients who had received background-targeted drug therapy, macitentan significantly improved the primary endpoint by 38% (Pulido et al., 2013). Furthermore, in a hemodynamic sub-study of SERAPHIN, macitentan therapy was associated with the improved cardiac index, right atrial pressure, mean pulmonary artery pressure (mPAP), PVR, and N-terminal pro-brain natriuretic peptide (NT-proBNP) levels (Galie et al., 2017). The multicenter randomized controlled trial phase III Macitentan in Eisenmenger Syndrome to Restore Exercise Capacity study revealed that NT-proBNP levels and PVR decreased in patients with Eisenmenger syndrome who received macitentan compared with those who received placebo, although it did not show superiority over placebo on the primary endpoint of change from baseline to week 16 in the 6-min walk test (Gatzoulis et al., 2019).

With the wide medications options available to physicians managing PAH patients, there is often a solid pharmacological and clinical reason for patients switching to a newer agent from ambrisentan to reduce adverse effects (AEs) or achieve greater efficacy. Additionally, ambrisentan has not been fully covered by insurance in China since 2019, and the financial burdens of the patient and the health care system may be reduced by at least 60% with macitentan therapy. Given these benefits, it is anticipated that many patients and clinicians will choose to transition from ambrisentan to macitentan therapy. However, clinical experience in switching patients from ambrisentan to macitentan is rarely reported. Although these drugs share a common mode of action, there are likely differences in individual patients’ responses in terms of clinical effectiveness and tolerance. Furthermore, side effects, such as liver toxicity and drug interactions differ between the two ERA. Moreover, although there is a pharmacokinetic basis for different efficacy of macitentan, no trials have been performed to directly compare the efficacy and safety of the two ERAs.

Since physicians and patients may consider converting from ambrisentan to macitentan, this open-label, prospective study (ChiCTR2000034898), was carried out to resolve whether such transition could be reasonably and safely accomplished, the proportion of patients that successfully transition, the frequency and severity of AEs during and after transition, and various clinical parameters. The study was also aimed at providing a reference for cautious, carefully supervised, safe, and efficient therapeutic transitions within the endothelin pathway and to monitor the process of this approach.

## Materials and Methods

### Study Design

This trial was a 12-month follow-up, real-world, prospective, open-label cohort study. The main objective was to evaluate the safety, tolerability, and efficacy of replacing ambrisentan with macitentan therapy in patients with PAH using a rapid switch protocol. Additionally, the impact of the change in therapy on treatment satisfaction was assessed by the patients’ quality of life (QOL) questionnaire. The trial received approval from the ethics committee at Second Xiangya Hospital of Central South University and was undertaken under the Declaration of Helsinki. All enrolled subjects provided informed consent before the entry into the study. The trial was registered at the Chinese Clinical Trial Registry (www.chictr.org.cn; No. ChiCTR2000034898).

### Selection Process and Criteria

The study consisted of a screening period, followed by a transition phase (the first 1 month), after which patients proceed to long term follow-up phase (12 months). All PAH patients voluntarily tried to change the ERA to macitentan due to the unsatisfactory treatment effect of ambrisentan, the desire to obtain better drug treatment effects, or the desire to reduce the financial burden. Patients who wish to switch must also undergo a rigorous assessment by doctors to ensure they are suitable for drug transition. Patients were enrolled in the study if they met the following criteria: 1) aged over 18 years; 2) stable for more than 3 months, defined as no worsening of WHO FC within 3 months, no clinical evidence of heart failure, and diuretic dose increase less frequently than once per month; 3) PAH diagnosis was confirmed by right heart catheterization: mPAP ≥ 25 mmHg; pulmonary capillary wedge pressure ≤ 15 mmHg; PVR ≥ 3 Wood units; 4) ambrisentan used for at least 6 months before transition, and a stable dose for no less than 3 months; 5) 6-min walk distance (6 MWD) of ≥250 m; 6)function class is no worse than WHO FC III; 7)women required to use at least one method of contraception during the study; 8) idiopathic PAH (IPAH), PAH associated with connective tissue disease (CTD-PAH), or PAH due to congenital heart disease (CHD-PAH), drugs, or toxins use.

### Transitioning Protocol

The decision to change to macitentan was at the discretion of the clinicians and patients during clinic visits, and the strategy for changing drugs was negotiated with the patients. During the visit, the cardiologist explained the revised dosing scheme and potential AEs and ensured that patients understood how they could contact our team. On receipt of the medication, patients were guided to take their last dose of ambrisentan at the regular time of their transition date and to take a new ERA the next day. Patients were able to contact the clinical team *via* telephone whenever they had any questions. The patient could withdraw permission and discontinue participation at any point in the study.

### Outcome Measures

To evaluate the safety and efficacy of the transition, AEs and their relationship to the medication were monitored throughout the study, as well as any changes in concomitant therapies. However, to facilitate analyses, we reported the absolute number and incidence of AEs at baseline and months 1, 3, 6, and 12 for the statistical analysis stage. Serum levels of transaminases and hemoglobin were measured after transition at months 1, 3, 6, and 12.

To assess whether patients maintained clinical stability, efficacy assessment was conducted at baseline and months 6 and 12 and included a physical examination, vital signs, 6 MWD, WHO FC, NT-proBNP, echocardiography, and REVEAL 2.0 risk score calculator (Benza et al., 2015b). Absolute change in 6 MWD from baseline, and the proportion of patients with an increased (>15%), maintained (±15%), and decreased (>15%) 6 MWD from baseline were calculated. A cut-off value of 15% was selected to take account of the instability of the 6 MWD results. Echocardiographic parameters measured the diameter of the right atrium (RA) and ventricle, the diameter of the pulmonary artery (PA), the tricuspid annular plane systolic excursion (TAPSE), the systolic velocity of the tricuspid annulus (S′), the right ventricular area change rate (RVFAC), and the estimated pulmonary artery systolic pressure (sPAP). The echocardiography was elucidated by cardiologists blinded to the study procedures. Risk stratification was assessed by the REVEAL 2.0 risk score calculator. The correspondence between the low-, intermediate-, and high-risk groups, as defined by the REVEAL 2.0 calculator, were as follows: low risk = REVEAL score ≤ 6, intermediate risk = REVEAL score 7 and 8, and high risk = REVEAL score ≥ 9 (Hoeper et al., 2017). Preceding right heart catheterizations were necessitated to confirm the diagnosis of PAH and were collected as a baseline characteristic but were not used to monitor patients during the transition. QOL was assessed by a medicines questionnaire (Lopez et al., 2021) at baseline and at the final follow-up to evaluate patients’ perceptions of treatment effectiveness, convenience, and satisfaction.

### Statistical Analysis

Quantitative data (age, 6 MWD, NT-proBNP, and echocardiography parameters) were described as means and standard deviations (normal or approximately normal distribution) or medians and interquartile ranges (IQR) (non-normal distribution). Normally distributed data were compared using a one-way analysis of variance for repeated measurement data, and non-normal distributions were compared using Friedman’s M test. Qualitative data (AEs) and ranked data (WHO FC and REVEAL score) were described by the number and percentage. Ranked data were compared using Friedman’s M test, and qualitative data were compared using Cochran’s Q test. Differences with a *p*-value of < 0.05 were considered significant.

## Results

### Patients’ Clinical Characteristics

**FIGURE 1:**
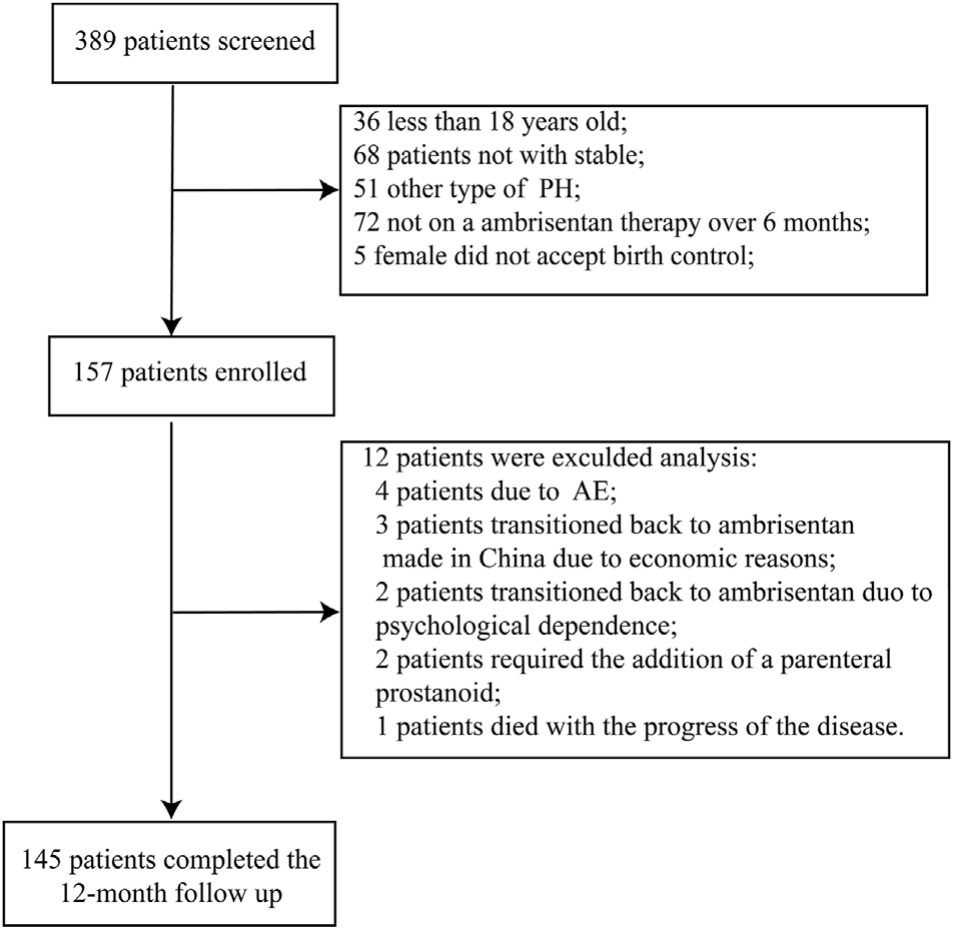
Flow chart of patients selection.

389 PAH patients were screened for participation in the study, 232 were excluded; 36 were less than 18 years of age; 68 were unstable; 51 had non-group 1 Pulmonary Hypertension; 72 were not on ambrisentan therapy for at least 6 months; 5 female patients were not willing to use any contraception (details in Figure 1). Patients were mostly female (74.5%) and WHO FC II at baseline (58.6%; details in Table 1). The majority of patients were CHD-PAH (60.7%), followed by IPAH (22.1%) and CTD-PAH (14.4%). Most patients (87.6%) were receiving a PDE-5i, or riociguat in addition to ambrisentan at baseline. Patients had been receiving ambrisentan for a median of 1.5 years (IQR, 1.0–2.9 years) before participating in the study. Sixty-one patients (42.1%) were receiving 10 mg once per day, whereas eighty-four patients (57.9%) were receiving 5 mg once daily.

**TABLE 1:** Baseline characteristics.

Characteristic	All patients (*N* = 145)
Age, years^a^	32.0 (26.0, 42.0)
Sex, n (%)	
Female	108 (74.5)
Male	37 (25.5)
Duration of ambrisentan treatment before transition, years^a^	1.5 (1.0, 2.9)
mPAP, mmHg^b^ ^,^ ^c^	61.4 ± 18.6
PVR, Wood^a^ ^,^ ^c^	14.4 (8.0, 22.1)
mRAP, mmHg^b^ ^,^ ^c^	10.0 ± 5.8
mRVP, mmHg^b^ ^,^ ^c^	41.7 ± 12.4
Etiology of PAH, n (%)	
Idiopathic	32 (22.1)
Drug or toxin-induced	4 (2.8)
Associated with connective tissue disease	21 (14.4)
Associated with congenital heart disease	88 (60.7)
Ambrisentan dose, mg, QD	
5 n (%)	84 (57.9)
10 n (%)	61 (42.1)
WHO FC, n (%)	
II	85 (58.6)
III	60 (41.4)
BMI (kg/m2)^b^	21.0 ± 3.2
PAH medications in addition to taking ambrisentan, n (%)	
Monotherapy	18 (12.4)
PDE-5i	122 (84.1)
Riociguat	4 (2.8)
Riociguat and PDE-5i	1 (0.7)

a Data are described as medians and interquartile ranges (non-normal distribution).

b Data are described as means and standard deviations (normal or approximately normal distribution).

c Historical data from the most recent right heart catheterization before enrollment.

mPAP, mean pulmonary arterial pressure; PVR, pulmonary vascular resistance; mRAP, mean right atrial pressure; mRVP, mean right ventricular pressure; QD, once per day; PAH, pulmonary arterial hypertension; WHO FC, World Health Organization functional class; BMI, body mass index; PDE-5i, phosphodiesterase type 5 inhibitor.

### Treatment Transition

Of the 157 enrolled patients, all patients switched safely and stably from ambrisentan to macitentan within the first month of the study. All patients were stable when they ceased taking ambrisentan. At the time of conversion, all patients were on 10 mg of macitentan once daily, and most (145 of 157) participants were on a drug regimen throughout the study and completed the whole study from January 2020 to July 2021. One hundred and forty-five patients (92.4%) met all preplanned criteria for constant transition at month 12, and 12 patients were excluded from analyses due to early discontinuation. Of the 12 excluded patients, 2 (1.3%) patients transitioned to ambrisentan of Chinese manufacture for poor financial reasons; 3 (1.9%) transitioned back to ambrisentan due to psychological dependence; 4 (2.5%) female patients suspended macitentan due to AEs; 2 (1.3%) patients required the addition of a parenteral treprostinil due to disease progression; one patient died at the 37-week follow-up. The death was likely due to the sudden cessation of subcutaneous treprostinil therapy and was not considered related to the ERA treatment.

### Safety

One or more AEs occurred in 107 subjects (73.8%) during the trial. Table 2 compared AEs that occurred in only ≥ 5% of the study population. Most AEs commenced at the transition phase. The most frequent AEs were headache, peripheral edema, and anemia, consistent with ERA class effects. Despite the high incidence, most AEs were mild to moderate. Most AE profiles were similar before and after the drug transition, the exceptions being anemia and menstrual disorder after 6 months. The number of subjects with anemia gradually increased from 10 (6.9%) at baseline to a peak of 29 (20.0%) at 6 months (*p* < 0.001) and increased to 22 (15.2%) at 12 months (*p* = 0.024).

**TABLE 2:** Adverse events.

	Baseline	Month 1	Month 3	Month 6	Month 12	*p*-value
Patients with ≥ 1 AE, n (%)	67 (46.2)	73 (50.3)	73 (50.3)	65 (44.8)	64 (44.1)	0.431^ǂ^
Headache, n (%)	27 (18.6)	27 (18.6)	27 (18.6)	24 (16.6)	20 (13.8)	0.101^ǂ^
Peripheral edema, n (%)	32 (22.1)	33 (22.8)	35 (24.1)	29 (20.0)	28 (19.3)	0.467^ǂ^
Anemia, n (%)	10 (6.9)	14 (9.7)	20 (13.8)	29 (20.0)	22 (15.2)	0.004^ǂ^, 0.451^¶^, 0.06^§^, <0.001^ɠ^, 0.024^ɣ^, 0.187^ɥ^
Menstrual disorder, n (%)*	1 (1.3)	1 (1.3)	5 (6.3)	9 (11.4)	4 (5.1)	<0.001^ǂ^, 1.0^¶^, 0.057^§^, <0.001^ɠ^, 0.15 ^ɣ^
ALT, mean ± SD	18.3 ± 16.2	18.6 ± 16.2	19.7 ± 23.6	15.8 ± 12.27	15.9 ± 12.8	
AST, mean ± SD	22.9 ± 13.34	21.1 ± 13.1	26.3 ± 24.9	24.9 ± 22.6	25.1 ± 18.9	
ALT > ULN, n (%)	7 (4.8)	10 (6.9)	11 (7.6)	5 (3.4)	4 (2.8)	0.070^ǂ^
ALT > 3ULN, n (%)	1 (0.7)	0 (0)	1 (0.7)	0 (0)	0 (0)	0.558^ǂ^
AST > ULN, n (%)	13 (9.0)	10 (6.9)	8 (5.5)	16 (11)	7 (4.8)	0.051^ǂ^
AST > 3ULN, n (%)	1 (0.7)	0 (0)	2 (1.4)	3 (2.1)	1 (0.7)	0.316^ǂ^

*The proportion was calculated only in women of reproductive age (n = 79). ^ǂ^Five groups (Baseline, Month 1, Month 3, Month 6, and Month 12) of data were compared. Once the difference (*p* < 0.05) was significant, other groups were compared with baseline. ^¶^Month 1 versus Baseline, ^§^Month 3 versus Baseline, ^ɠ^Month 6 versus Baseline, ^ɣ^Month 12 versus Baseline, ^ɥ^Month 6 versus Month 12. AE: adverse event; ALT: alanine aminotransferase; AST: aspartate aminotransferase; SD: standard deviation; ULN: normalized upper limit.

Among the 112 transitioned female patients, 13 (15.7% of the 83 in reproductive age) patients experienced menstrual disorders, and the most common symptoms were menorrhagia and metrorrhagia. The number of subjects with menstrual disorder gradually increased from one (1.3%) to nine (11.4%) (*p* < 0.001) at month 6. Four women experienced severe anemia after 2–4 months of transition that was felt to be due to menorrhagia associated with the new therapy. The macitentan was discontinued on the patients’ request and iron supplementation was instituted for at least 3 months. No other patients discontinued the macitentan due to AEs.

### Clinical Outcomes

**FIGURE 2:**
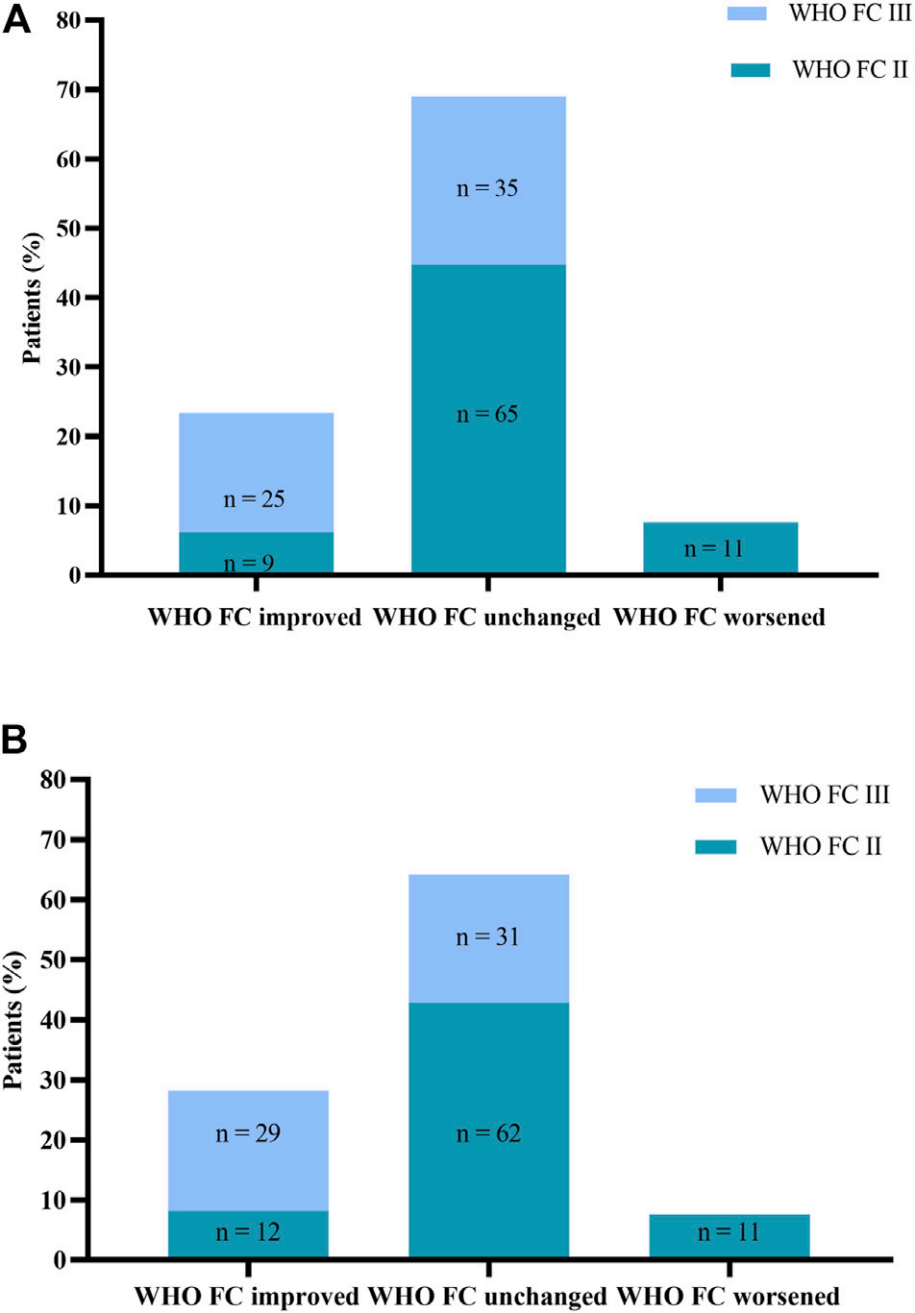
**(A)** Change in World Health Organization Functional Class (WHO FC). from baseline to Month 6. **(B)** Change in WHO FC from baseline to Month 12.

WHO FC, 6 MWD, and NT-proBNP assessments at baseline and months 6 and 12 are displayed in Table 3. From baseline to month 6, WHO FC stayed unchanged in 100 patients (69%) and ameliorated in 34 patients (23.4%; Figure 2A). Nine patients ameliorated from WHO FC II to FC I, and 25 (17.2%) from WHO FC III to FC II, whereas 11 patients deteriorated from WHO FC II to FC III. From baseline to month 12, WHO FC stayed unchanged in 93 patients (64.1%) and ameliorated in 41 patients (28.3%; Figure 2B). Twelve patients ameliorated from WHO FC II to FC I, 28 from WHO FC III to FC II, and one from WHO FC III to FC I, whereas 11 patients declined from WHO FC II to FC III. From months 6–12, WHO FC stayed unchanged in 137 patients (94.5%), and four patients each ameliorated from WHO FC II to FC I and from WHO FC III to FC II.

**TABLE 3:** Parameters at the baseline and months 6 and 12 assessments.

	Baseline	Month 6	Month 12	*p*-value
Echocardiography				
RA (mm)^ǂ^	40.33 ± 8.54	39.35 ± 8.90	39.09 ± 8.81	0.114^§^
RV (mm)^ǂ^	41.61 ± 9.06	40.23 ± 10.44	39.31 ± 9.42	0.006^§^, 0.056^ɠ^, 0.003^ɥ^
LA (mm)^ǂ^	32.17 ± 6.94	32.69 ± 6.21	33.18 ± 6.03	0.155^§^
LVEDD (mm)^ǂ^	40.39 ± 8.04	41.15 ± 7.50	40.98 ± 7.82	0.494^§^
PA (mm)^ǂ^	31.31 ± 5.91	31.23 ± 5.86	30.97 ± 5.54	0.802^§^
sPAP (mmHg)^ǂ^	86.10 ± 26.56	74.72 ± 21.23	77.14 ± 22.69	<0.001^§^, <0.001^ɠ^, <0.001^ɥ^, 0.566^ɣ^
TAPSE (mm)	13.98 ± 2.91^ǂ^	14.00 (12.00–17.75)^¶^	14.5 (13.00–17.75)^¶^	0.001^§^, 0.226^ɠ^, 0.001^ɥ^
S′ (cm/s)^¶^	11.00 (10.00–13.00)	13.00 (11.00–14.00)	13.00 (11.00–15.00)	<0.001^§^, 0.004^ɠ^, <0.001^ɥ^, 0.102^ɣ^
RVFAC^ǂ^	33.12 ± 7.65%	34.59 ± 7.81%	34.61 ± 8.06%	0.023^§^, 0.020^ɠ^, 0.022^ɥ^, 1.0^ɣ^
6 MWD (meters)^ǂ^	424.74 ± 82.34	435.92 ± 75.03	440.59 ± 77.57	0.004^§^, 0.016^ɠ^, 0.004^ɥ^, 0.714^ɣ^
NT-proBNP (pg/ml)^¶^	443.00 (140.90–1,143.5)	253.90 (110.0–1,009.00)	234.80 (108.95–939.50)	<0.001^§^, 0.045^ɠ^, <0.001^ɥ^, 0.005^ɣ^
S_p_O_2_	92.14	92.29	93.08	0.798
WHO FC, n (%)				<0.001^§^, 0.046^ɠ^, 0.008^ɥ^, 0.499^ɣ^
I	0	9 (6.2)	13 (9.0)	
II	85 (58.6)	90 (62.1)	90 (62.1)	
III	60 (41.4)	46 (31.7)	42 (29.0)	
Quality of life^ǂ^	74.10 ± 6.74	-	79.76 ± 13.66	<0.001^§^
REVEAL score, n (%)				<0.001^§^, <0.001^ɠ^, <0.001^ɥ^, 1.00^ɣ^
≤6	13 (9.0)	33 (22.8)	37 (25.5)	
7–8	42 (28.9)	58 (40.0)	51 (35.2)	
≥9	90 (62.1)	54 (37.2)	57 (39.3)	

ǂData are described as means and standard deviations (normal or approximately normal distribution). ^¶^Data are described as medians and interquartile ranges (non-normal distribution). ^§^Three groups (Baseline, Month 6, and Month 12) of data were compared. Once the difference (*p* < 0.05) was significant, other groups were compared with baseline. ^ɠ^Month 6 versus Baseline, ^ɥ^Month 12 versus Baseline, ^ɣ^Month 6 versus Month 12 (only when months 6 and 12 compared with baseline both were statistically significant, the two groups would be compared.). RA: right atrium; RV: right ventricle; LA: left atrium, LVEDD: left ventricular end-diastolic dimension; PA: pulmonary artery; TAPSE: tricuspid annular plane systolic excursion; S': systolic velocity of the tricuspid annulus; RVFAC: right ventricular area change rate; 6 MWD: 6-min walking distance; NT-proBNP, N-terminal pro-brain natriuretic peptide; S_p_O_2:_ pulse oxygen saturation; WHO FC: world health organization functional class.

**FIGURE 3:**
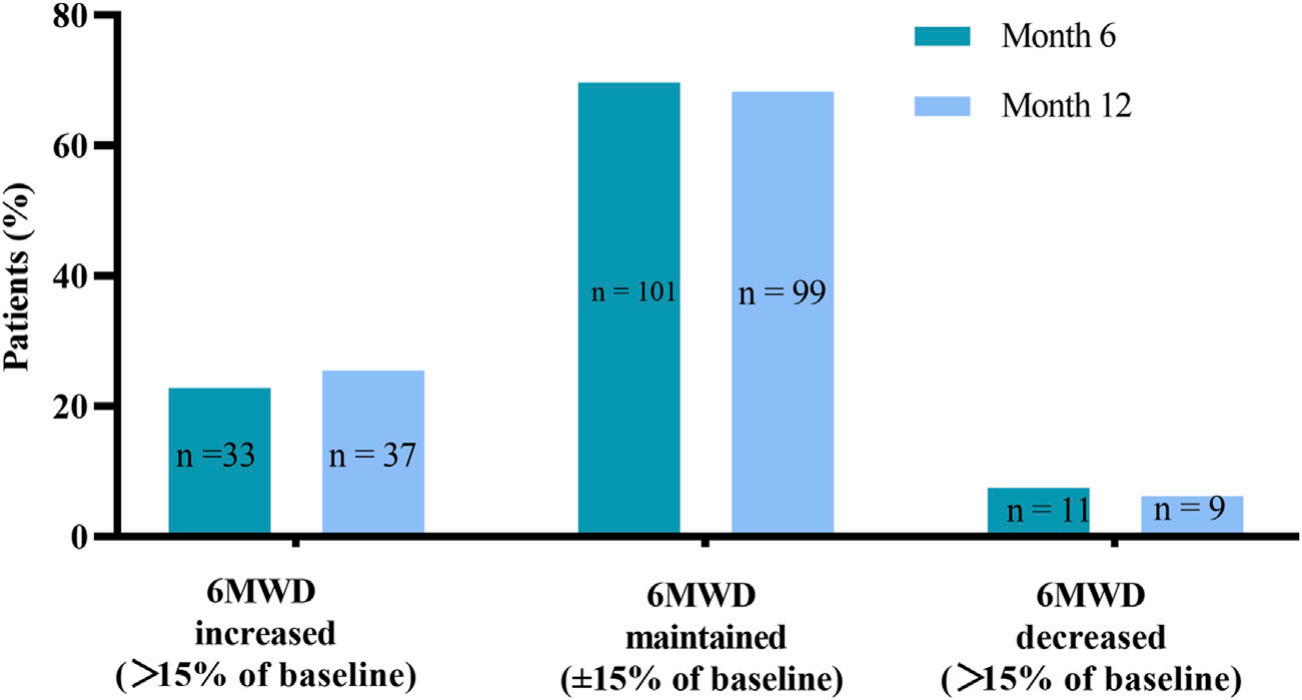
Change in 6-min walk distance (6 MWD) from baseline to Month 6 and Month 12.

The 6 MWD improved from 424.74 ± 82.34 m at baseline to 435.92 ± 75.03 m at month 6 (*p =* 0.016) and 440.59 ± 77.57 m at month 12 (*p =* 0.004). The median change in 6 MWD from months 6 to 12 was 1.00 m (IQR, −29.50 to 23.50 m; *p =* 0.714). In 101 (69.7%) patients, 6 MWD was maintained, and in 33 (22.8%) patients it was increased at month 6 (Figure 3). Eleven patients showed a decrease in 6 MWD from baseline at month 6. In 136 patients (93.8%), 6 MWD was either maintained or increased at month 12 (Figure 3). Nine patients showed a decrease in 6 MWD from baseline to month 12. Compared with month 6, only two patients showed a decrease in 6 MWD at month 12, others remained stable or increased. There was a clinically significant change in NT-proBNP levels from baseline to months 6 and 12 after the transition, the median decreased from baseline of 443.00 pg/ml (IQR, 140.90–1,143.5 pg/ml) to 253.90 pg/ml (IQR, 110.0–1,009.00 pg/ml; *p =* 0.045) and 234.80 pg/ml (IQR, 108.95–939.50 pg/ml; *p* < 0.001), respectively. The difference between month 6 and month 12 remained statistically significant (*p =* 0.005).

Regarding echocardiography parameters, no significant differences were found between diameters of the right atrium (RA), left atrium (LA), and left ventricular end-diastolic dimension (LVEDD) and pulmonary artery (PA) width. However, the basal diameter of the right ventricle (RV) was reduced from 41.61 ± 9.06 mm to 39.31 ± 9.42 mm (*p* = 0.003) at month 12, and sPAP was lowered from 86.10 ± 26.56 mmHg to 74.72 ± 21.23 mmHg (*p* < 0.001) at month 6 and to 77.14 ± 22.69 mmHg (*p* < 0.001) at month 12. TAPSE, S′, and RVFAC were used for the evaluation of RV function. TAPSE increased from 13.98 ± 2.91 mm to 14.5 (13.00–17.75) mm (*p* = 0.001) at month 12, the median change S′ from 11.00 (10.00–13.00) cm/s to 13.00 (11.00–14.00) cm/s (*p* = 0.004) at month 6 and to 13.00 (11.00–15.00) cm/s (*P* < 0.001) at month 12, and RVFAC from 33.12 ± 7.65%–34.59 ± 7.81% (*p* = 0.020) at month 6 and to 34.61 ± 8.06% (*p* = 0.022) at month 12, respectively. Of note, for those differences observed between baseline and months 6 or 12, no significant difference was found between months 6 and 12. The data on TAPSE, S’ and RVFAC was only available in 75 patients as most echocardiography did not focus on the parameters of RV.

**FIGURE 4:**
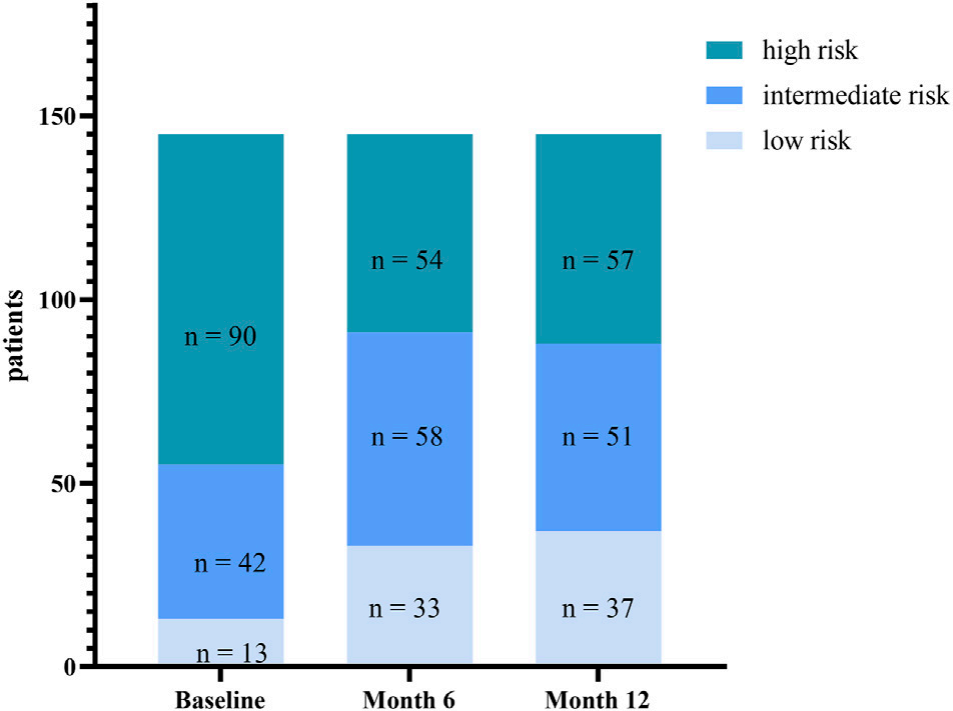
Change in risk stratification (assessed by REVEAL score 2.0) from baseline to Month 6 and Month 12.

The REVEAL 2.0 calculator was used to assess risk stratification at baseline and months 6 and 12 (Figure 4). At baseline, there were 13 patients with REVEAL risk scores ≤ 6, 42 patients with REVEAL risk scores between 7 and 8, and 90 patients with REVEAL risk scores ≥ 9. There were 33 patients with REVEAL risk scores ≤ 6 at 6 months after transition and 37 patients 12 months after the transition. There were 58 patients with REVEAL risk scores between 7 and 8 at 6 months after transition and 51 patients 12 months after the transition. There were 54 patients with REVEAL risk scores ≥ 9 at 6 months after transition and 57 at 12 months after the transition.

### Satisfaction and QOL

QOL scale was collected from 157 patients to assess treatment satisfaction. The data were not available for 12 patients who discontinued macitentan before month 12. QOL questionnaire contained therapeutic effects, the convenience of taking, impact on routine activities, overall satisfaction, and AEs (Lopez et al., 2021). The QOL scale score was 74.10 ± 6.74 at baseline and 79.76 ± 13.66 at month 12 (*p* < 0.001). There were no significant differences in items of AEs and convenience between time points, but there was a significant improvement in the activities of daily living and on the overall satisfaction.

## Discussion

The current evidence on the safety and efficacy of drug transition from one PAH-specific therapy to another includes the transition involving prostacyclin and its analogs (Hoeper et al., 2002; Gomberg-Maitland et al., 2005; Ewert et al., 2007; Rubenfire et al., 2007; Sitbon et al., 2007; Shlobin et al., 2012; Bourge et al., 2013; Minai et al., 2013; Shapiro et al., 2013; Tamura et al., 2013; Frantz et al., 2014; Frantz et al., 2015; Fukumoto et al., 2015; Lichtblau et al., 2015; Chakinala et al., 2017; Frost et al., 2019) or PDE-5i (Tay et al., 2008; Verlinden et al., 2020), but minimal data on the switch between ERA. A study reported that the switch from bosentan or sitaxentan to ambrisentan in patients with liver abnormalities is safe and can offer clinical improvement (McGoon et al., 2009). Two studies found that switching from sitaxentan to bosentan or ambrisentan is safe and tolerable as monitored by NT-proBNP levels, echocardiography, hemodynamic parameters, and side effects (Safdar, 2011; Fox et al., 2013). An observational study in 2018 showed that most PAH patients taking bosentan showed good safety and tolerance after replacement with ambrisentan or macitentan (Dawson et al., 2018). Moreover, a retrospective study in 2019 showed that after replacing bosentan or ambrisentan with macitentan, 6 MWD was increased and right ventricular function was improved in most patients (Cadenas-Menéndez et al., 2019). However, to date, no studies have especially explored the replacement of ambrisentan with macitentan. Thus, the evidence for the safety and efficacy of the transition from ambrisentan to macitentan in PAH patients needs to be considered.

In our study of 157 patients, the transition from ambrisentan to macitentan was successfully achieved in most patients, was well-tolerated, and without deterioration of clinical status. All enrolled patients (*n* = 157) continued to receive macitentan or at least 1 month after discontinuation of ambrisentan, and 145 patients complied with all prespecified criteria for the sustained switch. After study completion, 148 patients continued with macitentan. Patients were carefully selected and assessed throughout the study to prevent disease deterioration and to monitor for AEs.

Although most patients underwent some AEs, there were no differences in the overall incidence of AEs before and after the switch, but we need to take cautious of anemia and menstrual disorder in females. When taking ambrisentan, most AEs were peripheral edema and headache. After the transition, the incidence of peripheral edema and headache was not significantly changed, although there was a trend to less headache. Anemia and menstrual disorder, however, increased significantly. The proportion of patients with anemia at month 6 was higher in our study than that in the SERAPHIN trial (Pulido et al., 2013), and the most severe anemia occurred in females of reproductive age. Such anemia may not only be directly caused by macitentan but indirectly caused by metromenorrhagia associated with macitentan. Four female patients experienced severe anemia, shown by a decline in hemoglobin to 73, 75, 78, and 83 g/L, with associated menstrual disorder. They had to discontinue macitentan and receive iron therapy. After at least three months of therapy, hemoglobin normalized, and the menstrual disorder gradually recovered. Three of the women that stopped the macitentan did not have a recurrence of anemia after re-introduction of the macitentan and they remained stable. One of the women did not wish to restart the macitentan therapy. Although the mechanism by which macitentan causes anemia is unknown, our experience suggests that anemia can be associated with menstrual disorders in women. In addition, anemia can aggravate the symptoms of palpitation, chest tightness, and shortness of breath, resulting in patients’ noncompliance for prescribe. Thus, patients must monitor hemoglobin monthly. Once an abnormality is found, patients need to contact the doctors to evaluate it in time.

Regarding clinical stability, we used a strict definition and included multiple clinical parameters. Although several patients experienced a deterioration in WHO FC, 6 MWD, and NT-proBNP, most patients demonstrated stable or improved 6 MWD and WHO FC and thus, transitioned successfully. We observed 36 patients who moved from REVEAL 2.0 high-risk score to intermediate or low risk after the transition, and 33 patients maintained their condition 12 months later. Six months after the transition, the number of PAH patients (*n* = 91, 62.8%) in the low- or intermediate-risk groups reached a peak. Risk stratification score ameliorated, which indicates a better prognosis in our cohort of PAH patients. But our study was not professionally designed or powered to demonstrate superiority or non-inferiority over ambrisentan because of lacking a control group. The improvement observed may not be strongly reasoned to suggest that macitentan has better efficacy than ambrisentan. However, it is only suggested that patients taking macitentan do have a better effect, at least better than the baseline. Eleven patients experienced a worsening from baseline to month 6 in 6 MWD and nine patients at month 12. Eleven patients underwent deterioration in WHO FC, but none declined to WHO FC IV. The cause for the clinical worsening in a small proportion of patients was likely multifaceted and may include progressive deterioration of PAH together with the initiation of intravenous prostacyclin and the sudden withdrawal of the medication.

The hemodynamic parameters as assessed by echocardiography were comparable between pre-and post-transition, and there were no marked changes in the diameters of the RA, LA, LVEDD, and PA. However, we did observe significant decreases in the diameter of the RV and sPAP as well as increases in TAPSE, S′, and RVFAC from baseline to post-transition. These echo markers indicate improvement in RV function and may provide additional prognostic information in PAH (Augustine et al., 2018). The improvements in the mean diameter of RV and TAPSE were seen after 12 months, whereas the mean S’ and RVFAC significantly improved after 6 months. The improvement after transition may be attributable to optimized drug exposure by eliminating the drug interaction of ambrisentan or the possible advantage of dual ETA and ETB receptor antagonism. Nonetheless, these echocardiographic parameters were only obtained from 75 patients (52%), which may lead to bias due to the incomplete data and should only be considered exploratory in nature, and requires further validation with additional studies. Invasive hemodynamic measurements before and after the switch would also lend further strength to our data.

Treatment with macitentan may offer several benefits over ambrisentan therapy for Chinese patients. In China, although treatment decisions are based on the PAH guidelines, they are also largely influenced by patients’ financial situations (Zhang et al., 2011; Zhai et al., 2017). Both ambrisentan and macitentan are expensive in China. However, on April 1st, 2020, macitentan has been added to the list of medical insurance in Hunan province. After being reimbursed by 60–70% by the medical insurance, monthly out-of-pocket expenses of macitentan are about $187–249, while that of ambrisentan is $624 due to the withdrawal of medical insurance for the latter. Thus, ambrisentan therapy results in a substantial pill burden, which can increase non-adherence. Overall, the transition from ambrisentan to macitentan results in a cost-saving of approximately $14.5 per day. Hence, the transition enhanced overall treatment satisfaction and life happiness (QOL).

Our study is the first clinical study to demonstrate that converting from ambrisentan to macitentan can be completed safely in clinically stable patients. Limitations of this study are the open-label nature of the trial with no placebo or active comparator. In addition, the patient-reported QOL and treatment administration time data were inherently subjective, and long-term data were limited by missing data and might have been affected by completer bias of patients who may have discontinued the trial for reasons such as treatment dissatisfaction. As such, the clinical correlation of observed changes is not fully elucidated and the data should be cautiously interpreted. Despite these limitations, this study provides useful insight into the safety and efficacy of converting PAH patients from ambrisentan to macitentan therapy in a real-world clinical setting. The strengths of the study include the large size of the cohort, strict inclusion criteria, and 12-month follow-up.

## Conclusion

In summary, we have reported the sustained transition of a carefully selected cohort from ambrisentan to macitentan in PAH patients. These data indicate that the transition is safe and compared with baseline, the switch to macitentan therapy was associated with improvements in exercise capacity, NT-pro BNP, and right ventricular function (assessed by echocardiography) in the majority of PAH patients. However, the increase in the incidence of menstrual disorder and anemia requires careful monitoring and may lead to further intervention in younger women. We found that macitentan was associated with good compliance and maybe a good alternative to ambrisentan if the switch is required due to side effects or accessibility issues.

## Data Availability

The original contributions presented in the study are included in the article/Supplementary Material, further inquiries can be directed to the corresponding author.
