# Corrigendum: The Transition From Ambrisentan to Macitentan in Patients With Pulmonary Arterial Hypertension: A Real-Word Prospective Study

**DOI:** 10.3389/fphar.2022.891907

**Published:** 2022-04-11

**Authors:** Yusi Chen, Jun Luo, Jingyuan Chen, Eugene Kotlyar, Zilu Li, Wenjie Chen, Jiang Li

**Affiliations:** ^1^ Department of Cardiovascular Medicine, The Second Xiangya Hospital of Central South University, Changsha, China; ^2^ St. Vincent’s Hospital, Sydney, NSW, Australia

**Keywords:** pulmonary arterial hypertension, macitentan, ambrisentan, endothelin receptor antagonist, real-word study

In the original article, there was a mistake in Table 1 and in the Section **Results**, **Patients’ Clinical Characteristics** as published. “There were errors in the values for the ‘Ambrisentan dose’ as it was reported that one hundred and twenty-eight patients (88.3%) were receiving 10 mg once per day, whereas seventeen patients (11.7%) were receiving 5 mg once daily. This was corrected to sixty-one patients (42.1%) were receiving 10 mg once per day, and eighty-four patients (57.9%) were receiving 5 mg once daily*.*” The corrected Table 1 appears below and the text in the Section **Results**, **Patients’ Clinical Characteristics** has been updated.

**TABLE 1:** Baseline characteristics.

Characteristic	All patients (*N* = 145)
Age, years^a^	32.0 (26.0, 42.0)
Sex, n (%)	
Female	108 (74.5)
Male	37 (25.5)
Duration of ambrisentan treatment before transition, years^a^	1.5 (1.0, 2.9)
mPAP, mmHg^b^ ^,^ ^c^	61.4 ± 18.6
PVR, Wood^a^ ^,^ ^c^	14.4 (8.0, 22.1)
mRAP, mmHg^b^ ^,^ ^c^	10.0 ± 5.8
mRVP, mmHg^b^ ^,^ ^c^	41.7 ± 12.4
Etiology of PAH, n (%)	
Idiopathic	32 (22.1)
Drug or toxin-induced	4 (2.8)
Associated with connective tissue disease	21 (14.4)
Associated with congenital heart disease	88 (60.7)
Ambrisentan dose, mg, QD	
5 n (%)	84 (57.9)
10 n (%)	61 (42.1)
WHO FC, n (%)
II	85 (58.6)
III	60 (41.4)
BMI (kg/m2) b	21.0 ± 3.2
PAH medications in addition to taking ambrisentan, n (%)
Monotherapy	18 (12.4)
PDE-5i	122 (84.1)
Riociguat	4 (2.8)
Riociguat and PDE-5i	1 (0.7)

a Data are described as medians and interquartile ranges (non-normal distribution).

b Data are described as means and standard deviations (normal or approximately normal distribution).

c Historical data from the most recent right heart catheterization before enrollment.

mPAP, mean pulmonary arterial pressure; PVR, pulmonary vascular resistance; mRAP, mean right atrial pressure; mRVP, mean right ventricular pressure; QD, once per day; PAH, pulmonary arterial hypertension; WHO FC, World Health Organization functional class; BMI, body mass index; PDE-5i, phosphodiesterase type 5 inhibitor.

The authors apologize for this error and state that this does not change the scientific conclusions of the article in any way. The original article has been updated.

